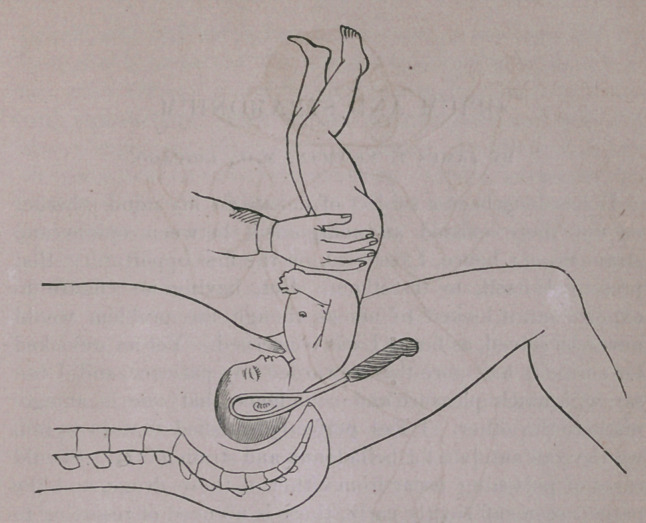# Dystocia, Originating in a Rare Position of the Fœtus

**Published:** 1868-12-01

**Authors:** H. Webster Jones

**Affiliations:** Accoucheur to Cook County Hospital


### DYSTOCIA, ORIGINATING IN A RARE POSITION OF THE FŒTUS.

BY H. WEBSTER JONES, ACCOUCHEUR TO COOK COUNTY HOSPITAL.

THE patient, Bridget D——, was a primipara, æt. 26, of robust constitution, and having arrived at term, was seized with active pains, at 7 P. M., October 18th, 1868.

The abdomen was unusually large; the os uteri high in the pelvis, and undilated, and no part of the fœtus was tangible per vaginam.

Through the abdominal walls the head could be felt, resting upon the right side of the pelvic brim. External manipulation, between pains, succeeded in effecting only its temporary dislodgement.

During the night, pains were frequent and severe, attended by vomiting and restless apprehension.

At 4 A. M., October 19th, the os uteri was found to be fully dilated, with the membranes protruding; the head was discoverable per vaginam, located as before mentioned, and resisting all effort to engage it in the superior strait.

Pulse 110; tongue dry and parched.

At 5 A. M. the patient was anæsthetized, the membranes ruptured (there was an excessive discharge of liquor amnii), and the hand fully introduced within the uterine mouth.

The occiput was found to occupy the right sacro-iliac fossa, the forehead resting upon the pubis, at the right of the symphysis; the head was strongly extended, the back arched upward, and to the extreme left was a foot (apparently the right), with the heel directed inward. Between head and foot was a wide space, in which the hand could be freely moved, occupied solely by a pulsating loop of the umbilical cord.

Efforts at flexing and votating the head proved futile; neither was it possible to draw down the foot to any considerable extent. Dr. Jones was sent for at 8 A. M.

Thus far our able house-physician, Dr. N. Senn, narrates the case.

Upon my arrival, at noon, the patient was found to be in an irritable condition, her pains severe; pulse one hundred and twenty; tongue dry; otherwise apparently in *statu quo.*

Chloroform being now administered by Dr. Senn, I proceeded to verify the diagnosis already made, and, with additional assistance from Drs. D. S. Root and Miller, to deliver the patient by such method as might be most promising.

The accompanying diagram illustrates very nearly the position then occupied by the fœtus. Agreeing in essentials with the statements before made, the degree of extension of the head present, taken coincidentally, with the posterior relations of the vertex secured, to favor podalic version rather than a forceps delivery.

Both feet were now firmly grasped, and an effort made, aided by external manipulation, to rotate the body on its transverse axis, in elevating the head and depressing the pelvic extremity, as well as, longitudinally, to turn the back of the child toward the front of the mother. This complex attempt was successful only in the substitution of an anterior for a posterior rotating of the trunk.

The descent of the fœtus upon the latter procedure, was as represented in figure second.

Rotation to the right being still continued, the left shoulder was first to appear at the vulva, the arms having already been brought down.

A difficulty here occurred, in the failure of the head to follow the line of rotation indicated. It was arrested, as shown in figure third, the face looking toward the right groin, with so much of flexion, as that the chin was not caught upon the pelvic brim, yet apparently immovable as regards traction by the trunk, by the fingers within the mouth, or by external pressure above the pubis.

The fœtal pulse now flagging, immediate resort to the forceps was determined upon.

It was impossible to adjust the female (right) blade upon the anterior aspect of the face, so that rotation into the sacral hollow could be effected.

Delivery of the head was partly accomplished, as portrayed in figure fourth, the mental extremity of its long diameter finding easy exit beneath the pubic arch.

Life was extinct, the operation having occupied three quarters of an hour, and the cord subjected, meantime, to a pressure almost constantly hazardous.

The mother recovered without untoward symptoms.

The position of the child, as described in the early stage of labor, could have been possible only in the presence of a large excess of amniotic fluid.

The sudden and severe onset of uterine contractions, probably conduced to its maintenance, their immediate effect upon the fœtal arch being to separate feet and head more widely, and deter either extremity from the superior strait.

Such a condition is certainly very rare, and is believed to be hitherto unrecorded.

## Figures and Tables

**Figure f1:**
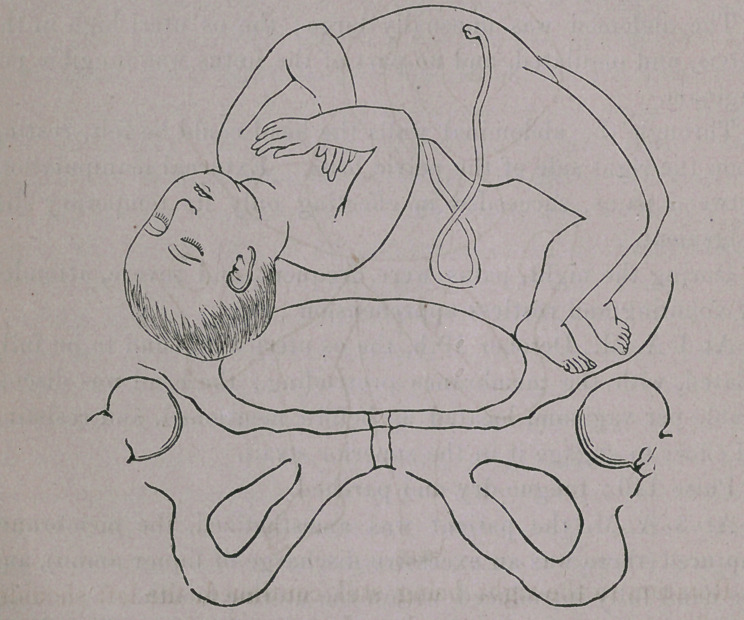


**Figure f2:**
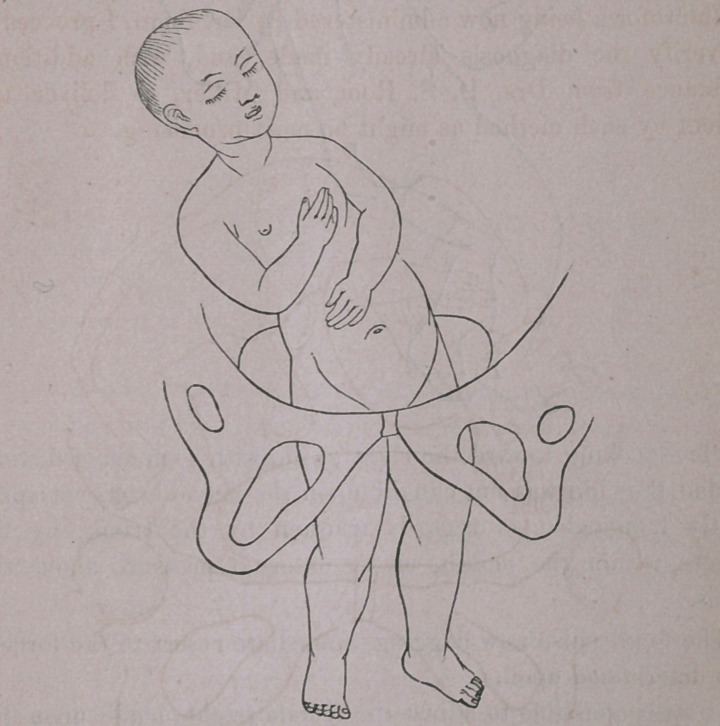


**Figure f3:**
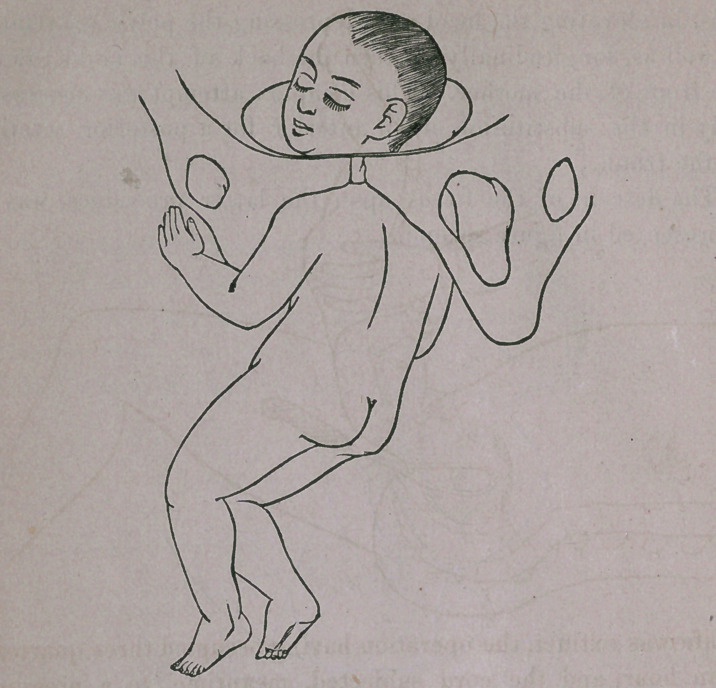


**Figure f4:**